# Serum Amyloid A Induces Toll-Like Receptor 2-Dependent Inflammatory Cytokine Expression and Atrophy in C2C12 Skeletal Muscle Myotubes

**DOI:** 10.1371/journal.pone.0146882

**Published:** 2016-01-19

**Authors:** Samantha L. Passey, Steven Bozinovski, Ross Vlahos, Gary P. Anderson, Michelle J. Hansen

**Affiliations:** 1 Lung Health Research Centre, Department of Pharmacology and Therapeutics, University of Melbourne, Parkville, Melbourne, Victoria, Australia; 2 School of Health and Biomedical Sciences, RMIT University, Bundoora, Melbourne, Victoria, Australia; McGill University, CANADA

## Abstract

**Background:**

Skeletal muscle wasting is an important comorbidity of Chronic Obstructive Pulmonary Disease (COPD) and is strongly correlated with morbidity and mortality. Patients who experience frequent acute exacerbations of COPD (AECOPD) have more severe muscle wasting and reduced recovery of muscle mass and function after each exacerbation. Serum levels of the pro-inflammatory acute phase protein Serum Amyloid A (SAA) can rise more than 1000-fold in AECOPD and are predictively correlated with exacerbation severity. The direct effects of SAA on skeletal muscle are poorly understood. Here we have examined SAA effects on pro-inflammatory cachectic cytokine expression (IL-6 and TNFα) and atrophy in C2C12 myotubes.

**Results:**

SAA increased IL-6 (31-fold) and TNFα (6.5-fold) mRNA levels compared to control untreated cells after 3h of SAA treatment, and increased secreted IL-6 protein at 24h. OxPAPC, a dual TLR2 and TLR4 inhibitor, reduced the response to SAA by approximately 84% compared to SAA alone, and the TLR2 neutralising antibody T2.5 abolished SAA-induced expression of IL-6, indicating that SAA signalling in C2C12 myotubes is primarily via TLR2. SAA also reduced myotube width by 10–13% and induced a 2.5-fold increase in the expression of the muscle atrophy gene Atrogin-1, suggesting direct effects of SAA on muscle wasting. Blocking of TLR2 inhibited the SAA-induced decrease in myotube width and Atrogin-1 gene expression, indicating that SAA induces atrophy through TLR2.

**Conclusions:**

These data demonstrate that SAA stimulates a robust pro-inflammatory response in skeletal muscle myotubes via the TLR2-dependent release of IL-6 and TNFα. Furthermore, the observed atrophy effects indicate that SAA could also be directly contributing to the wasting and poor recovery of muscle mass. Therapeutic strategies targeting this SAA-TLR2 axis may therefore ameliorate muscle wasting in AECOPD and a range of other inflammatory conditions associated with loss of muscle mass.

## Introduction

Peripheral muscle wasting is experienced by approximately one-third of Chronic Obstructive Pulmonary Disease (COPD) patients [1]. Loss of muscle mass and function has a profound impact on the quality of life for COPD patients, leading to reduced independence and increased use of healthcare resources [2]. Studies investigating the link between mortality and mid-thigh cross sectional area [3], fat-free mass [4] and quadriceps strength [5] have demonstrated muscle wasting to be an independent predictor of mortality for COPD, particularly in patients with low lung function (Forced expiratory volume in 1s, FEV_1_ of <50%).

Acute exacerbations of COPD (AECOPD), usually caused by viral or bacterial infections, are characterised by a worsening of COPD symptoms including shortness of breath and increased sputum production, often requiring alteration in the medical management of the disease and/or hospitalisation. In addition to pulmonary effects, exacerbations also impact skeletal muscle health and function, further contributing to muscle wasting. Hospitalised patients with AECOPD displayed lower mean quadriceps peak torque at both day 3 and day 8 of hospitalisation compared to stable COPD outpatients or sedentary elderly controls [6]. Patients recover muscle strength slowly and only partially, reflecting an inverse correlation between the frequency of exacerbations and both respiratory and limb muscle strength [7, 8]. For example, patients experiencing five AECOPD in the preceding year showed a handgrip strength of only 42.8% of predicted, compared to 89.6% of predicted handgrip strength in COPD patients experiencing no exacerbations [7].

During AECOPD systemic inflammation has been shown to dramatically increase, with the production of pro-inflammatory cytokines such as IL-6 and TNFα promoting expression of acute phase proteins that are rapidly generated as part of the inflammatory response. The acute phase protein Serum Amyloid A (SAA) is dramatically upregulated during AECOPD, and has been shown to be a sensitive biomarker for the prediction of exacerbation severity in COPD patients [9]. During the acute phase response, plasma levels of SAA can increase 1000-fold, thereby constituting a major circulating acute phase reactant.

In addition to COPD, elevated SAA levels have been measured in other inflammatory conditions, many of which are associated with muscle wasting, including critical illness myopathy [10], rheumatoid arthritis [11] and lung cancer [12]. The major source of circulating SAA is the liver, and there is emerging evidence for accumulation of SAA in peripheral organs in chronic inflammatory diseases including the lungs of COPD patients [13] and the synovial fluid of arthritic patients [11]. In addition we have shown that in experimental models, cigarette smoke, influenza A virus and bacterial lipopolysaccharide (LPS) increase SAA transcript expression in the lungs [13]. SAA expression is also increased in the quadriceps and gastrocnemius muscles of mice with cancer cachexia [14].

A number of studies have demonstrated a relationship between systemic inflammation and muscle health, and pro-inflammatory cytokines IL-6 and TNFα are known to induce muscle atrophy in animal models [15, 16]. In COPD patients muscle strength and exercise endurance are inversely related to the levels of systemic inflammatory proteins including C-reactive protein (CRP), IL-6 and CXCL8 [6, 17–19]. Emerging evidence suggests links between SAA levels and muscle atrophy in other disease models. SAA expression is elevated in response to Angiotensin II infusion in mice, and evidence suggests that increased circulating SAA acts in synergy with elevated IL-6 to promote skeletal muscle wasting in this model, implicating SAA in the pathology of skeletal muscle atrophy [20].

Despite emerging evidence suggesting effects of elevated SAA on skeletal muscle wasting in disease models, little is known about the direct effects of SAA on skeletal muscle. To address this we sought to determine if SAA could be contributing to changes in muscle function by directly assessing the effects of SAA treatment on *in vitro* cultured C2C12 myotubes.

## Materials and Methods

### Cell Culture

Murine myoblast C2C12 cells (American Type Culture Collection via Cryosite, NSW, Australia) were cultured in growth medium (GM) consisting of high glucose DMEM supplemented with 20% foetal bovine serum, and 1% penicillin (100 units/ ml)/streptomycin (100 μg/ ml). To induce differentiation, confluent monolayers of C2C12 cells were cultured in differentiation medium (DM) consisting of DMEM supplemented with 2% horse serum and 1% Penicillin-streptomycin. After 72h in differentiation media C2C12 myotubes were treated with recombinant human SAA (Peprotech, Abacus ALS, Australia) at concentrations ranging from 0.1–10μg/ml for 2-48h. In experiments assessing the inhibition of the SAA response a concentration of SAA was used that stimulated a significant but submaximal response for the outcome being measured. SAA is well conserved across species [21]. Acute (2-3h) time points were chosen to investigate early transcriptional effects of SAA, whilst later time points, 24 and 48h, were used to study effects on protein secretion and atrophy responses in the myotubes, respectively.

For Polymyxin B experiments, SAA (3μg/ml) and Polymyxin B (20μg/ml, Invivogen, Life Research, Australia) were incubated in DM for 1 hour at room temperature prior to adding to cells. The fpr2 antagonist WRW4 (Invivogen) was added to cells in DM at 5.5 μg/ml for 20 minutes prior to treatment with SAA at 1μg/ml.

To inhibit TLR2/4 signalling, cells were incubated with the TLR2/4 inhibitor OxPAPC (oxidised 1-palmitoyl-2-arachidonyl-sn-glycero-3-phosphorylcholine, Invivogen, Life Research) at 30–100μg/ml for 30 minutes prior to the addition of SAA. The TLR2 neutralising antibody T2.5 (Invivogen) was incubated with cells at 10 or 20μg/ml for 1 hour prior to the addition of SAA.

### Immunohistochemistry

Differentiated C2C12 myotubes cultured on 13mm gelatin-coated glass coverslips were fixed in 10% Neutral Buffered Formalin (NBF) for 30 minutes then washed in PBS. Following blocking in PBS containing 5% horse serum and 0.2% Triton X-100 for 15 minutes, coverslips were incubated for 1 hour with Rabbit anti-desmin (Abcam, Australia) at a 1:200 dilution followed by 1 hour with goat FITC anti-rabbit (Abcam). Myotube widths were quantified from microscope images, using a similar method to that reported by others [22–24]. Ten images were captured from randomly selected fields of view for each treatment group using a Zeiss Axio Observer Z1 inverted fluorescence microscope. The width of each myotube was calculated as the mean of three width measurements at points at least 50μm apart along the length of the myotube. Myotubes that were overlapping with others or had indistinguishable boundaries were excluded. For each treatment group 220–330 myotubes were measured over three independent experiments.

### Quantitative Real-time PCR

Total mRNA was isolated from C2C12 myotube cultures from 24-well plates using the RNEasy Plus kit according to the manufacturer’s instructions (Qiagen). RNA was quantified using a Nanodrop 1000 (Nanodrop, Thermofisher Scientific, Australia).Gene expression was measured by qRT-PCR using the Taqman PCR system (Applied Biosystems, Thermofisher Scientific, Australia). Taqman primers and probes were purchased as validated PCR assays for IL-6 (Mm00446190_m1), TNFα (Mm00443258_m1), fpr2 (Mm00484464_s1), TLR2 (Mm00442346_m1), TLR4 (Mm00445273_m1), CD36 (Mm01135198_m1) and Atrogin-1 (Mm 00499518_m1). Expression of mRNA for genes of interest was normalised to 18S rRNA (Taqman Eukaryotic 18S rRNA endogenous control, Applied Biosystems). Quantitative RT-PCR reactions were performed using Taqman Fast Advanced Master Mix using the 7900HT Fast real-time PCR machine (Applied Biosystems). Samples were analysed in triplicate. Gene expression was analysed using relative quantification and the ΔΔCt method [25], expression levels were normalised to 18S.

In some experiments, PCR products from triplicate reactions were pooled and analysed by agarose gel electrophoresis using 2% agarose gel. DNA was visualised using SYBR-safe (Thermofisher Scientific) and molecular weight confirmed with a 100bp DNA ladder (New England Biolabs, Genesearch Pty Ltd. Arundel, QLD, Australia). Gels were imaged using a Bio-Rad Gel-doc XR imaging system (Bio-Rad, Gladesville, NSW, Australia).

### Measurement of secreted IL-6 by ELISA

Secreted IL-6 was measured in cell culture supernatants using the Platinum mouse IL-6 ELISA kit (eBioscience, Jomar Bioscience, South Australia) according to the manufacturer’s instructions. TNFα was measured by ELISA with the mouse TNFα Duoset ELISA development kit (R&D Systems, BioScientific Pty. Ltd. Australia).

### Statistical Analysis

All data presented are the mean ± SEM of three independent experiments (n = 3). Statistical analysis of data was performed using GraphPad Prism 6 (GraphPad Software, California, USA). Experiments with only two groups were analysed using Students t-test. Datasets with multiple groups were analysed by One-way ANOVA, and data from time course experiments with multiple groups were analysed by Two-way ANOVA. A Sidak’s multiple comparison *post-hoc* test was used to compare the means between treatment groups where a significant difference in the group means was detected by ANOVA. In all cases, probability values of less than 0.05 (p<0.05) were considered statistically significant.

## Results

### SAA causes upregulation of pro-inflammatory cytokine expression in C2C12 myotubes

**Fig 1:**
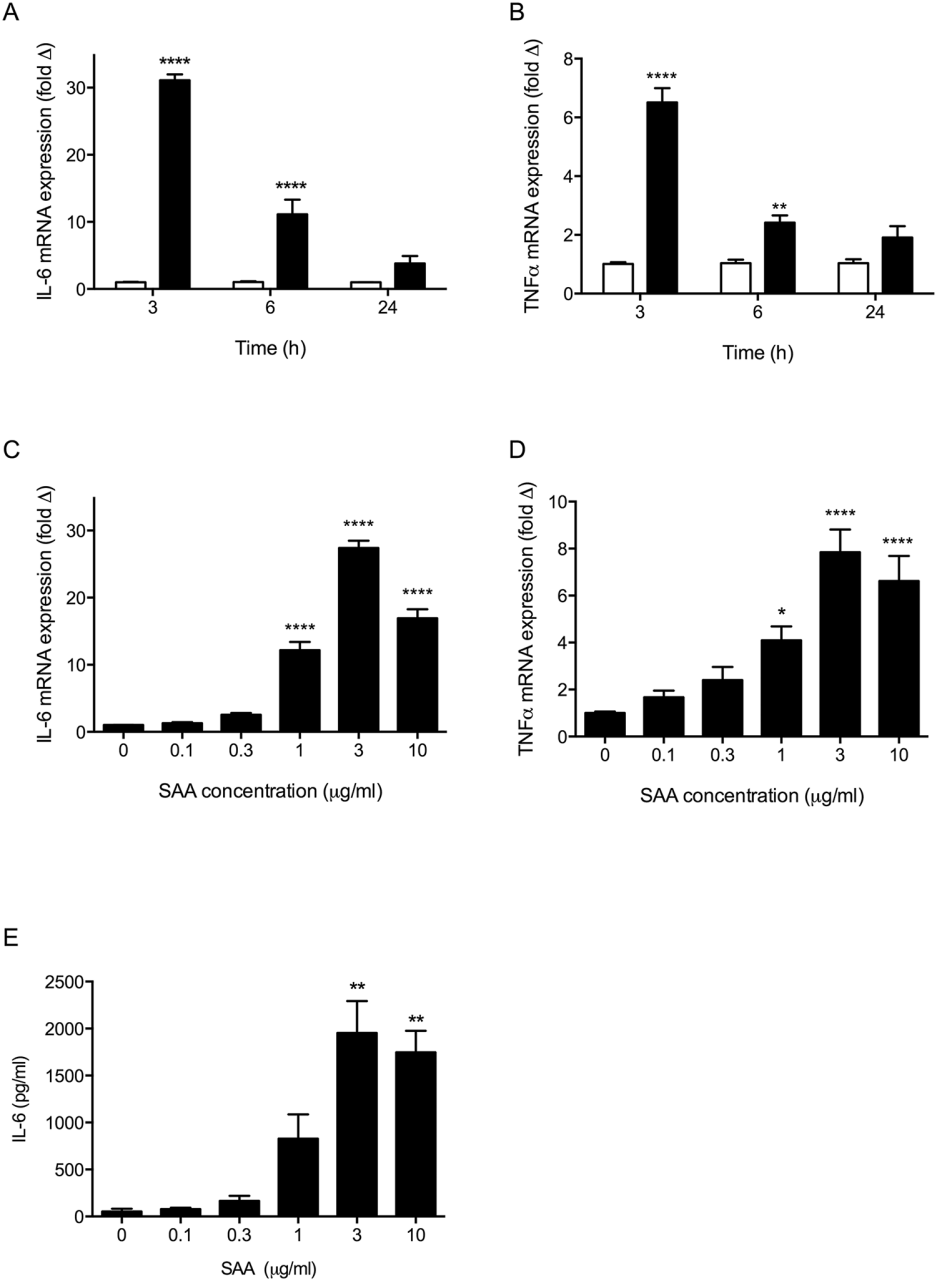
C2C12 myotubes express pro-inflammatory cytokines IL-6 and TNFα in response to SAA. (A) IL-6 and (B) TNFα mRNA expression after 3, 6 and 24h of SAA stimulation (10μg/ml). Solid bars = SAA treated, open bars = control. mRNA level normalised to 18S rRNA levels and expressed relative to control 3h samples, **** p<0.0001, ** p<0.01 *vs*. control 3h sample. (C) IL-6 and (D) TNFα mRNA expression levels after 3h of stimulation with varying concentrations of SAA from 0.1 to 10 μg/ml. mRNA level normalised to 18S rRNA levels and expressed relative to control untreated samples, **** p<0.0001 and * p< 0.05 vs 0μg/ml SAA. (E) IL-6 protein measured by ELISA in cell culture supernatants after 24h of treatment with SAA at 0–10μg/ml, ** p<0.01 *vs*. control 0 μg/ml SAA. All data shown are mean ± SEM for n = 3 independent experiments.

The effect of SAA treatment on muscle cells was assessed using the mouse C2C12 cell line [26], which differentiates into multinucleate myotubes and is well established as an *in vitro* model of skeletal muscle [27]. To determine the effect of SAA on pro-inflammatory gene expression in C2C12, differentiated myotubes were treated with 10μg/ml SAA and mRNA was harvested after 3, 6 and 24h of SAA treatment. Gene expression for the pro-inflammatory cytokines IL-6 and TNFα was analysed by qRT-PCR. SAA treatment resulted in a dramatic and significant upregulation in IL-6 mRNA expression after both 3h and 6h of SAA (p<0.0001 SAA vs. control untreated cells at 3h), returning to baseline at 24h (Fig 1A). A similar trend was also seen for TNFα, with highest expression at 3h (Fig 1B, p<0.0001 SAA vs. control at 3h).

Expression of IL-6 and TNFα in response to SAA treatment was also shown to be concentration dependent. Both IL-6 and TNFα mRNA expression were significantly increased at 1, 3 and 10μg/ml, with peak expression seen in myotubes stimulated with 3μg/ml SAA (p<0.0001, Fig 1C and 1D).

SAA also induced a concentration dependent increase in secreted IL-6 protein measured by ELISA in cell culture supernatants following 24h of SAA treatment, with peak IL-6 protein detected in cells treated with 3μg/ml SAA (p<0.01, Fig 1E). Despite a concentration dependent increase in TNFα mRNA expression, the level of secreted TNFα protein in SAA-treated cell culture supernatants was below the limit of detection for our ELISA assay (31.1pg/ml). Similar observations have also been reported by others in C2C12 myotubes in response to LPS [28].

### Expression of SAA receptors in C2C12 myotubes

Given the known role of inflammatory cytokines in mediating muscle wasting, and the robust cytokine response we observed following SAA treatment, we sought to determine the receptor responsible for inducing these responses. A number of receptors for SAA have been described, including the formyl peptide receptor FPR2 (mouse nomenclature fpr2) [29], the Toll-like receptors TLR2 [30, 31] and TLR4 [32, 33] and the scavenger receptor CD36 [34]. We measured the expression of fpr2, TLR2, TLR4 and CD36 in C2C12 myotubes by qRT-PCR, both under basal conditions and in response to 3h of SAA stimulation.

**Fig 2:**
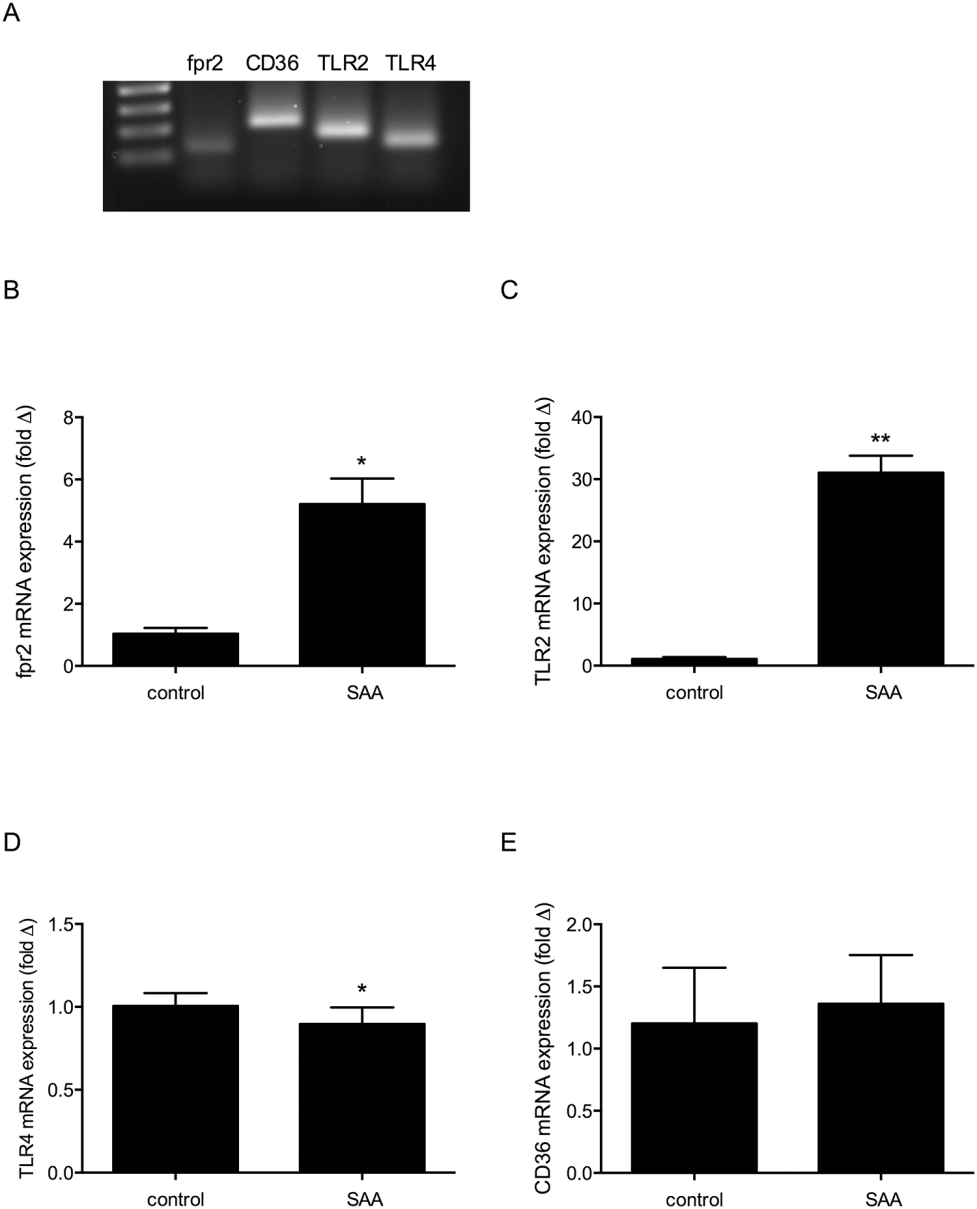
C2C12 myotubes express SAA receptors, and TLR2 and fpr2 expression increases following SAA stimulation. (A) Agarose gel electrophoresis showing RT-PCR bands from C2C12 myotube RNA samples. RT-PCR data measuring changes in mRNA levels for (B) fpr2, (C) TLR2, (D) TLR4 and (E) CD36 following 3h of treatment with SAA at 3μg/ml. mRNA levels normalised to 18s rRNA and expressed relative to control untreated sample for each receptor. Data shown are mean ± SEM from n = 3 independent experiments. Expression of each receptor was analysed using paired t-test, fpr2 * p<0.05), TLR2 ** p<0.01, TLR4 * p<0.05.

All four receptors were detected by qRT-PCR, and bands were visible by agarose gel electrophoresis (Fig 2A). CD36 and TLR4 were expressed with similar levels of abundance (raw qRT-PCR Ct values: CD36 25.18 ± 1.13, and TLR4 24.14 ± 1.56, n = 3), followed by TLR2 (Ct 27.52 ± 0.57, n = 3). Fpr2 expression, whilst detectable, was much less abundant than other receptors measured (mean Ct value 35.98 ± 0.54, n = 3).

Interestingly, expression of TLR2 was shown to dramatically increase in response to SAA treatment of cells (p<0.001, Fig 2B). Similarly whilst fpr2 was expressed at low levels under basal conditions, fpr2 mRNA increased significantly after 3h SAA stimulation (p<0.01, Fig 2B). A small but significant decrease was observed in TLR4 expression, and no change was observed in CD36 (p = 0.5512) following SAA stimulation.

### SAA Receptor blocking studies

**Fig 3:**
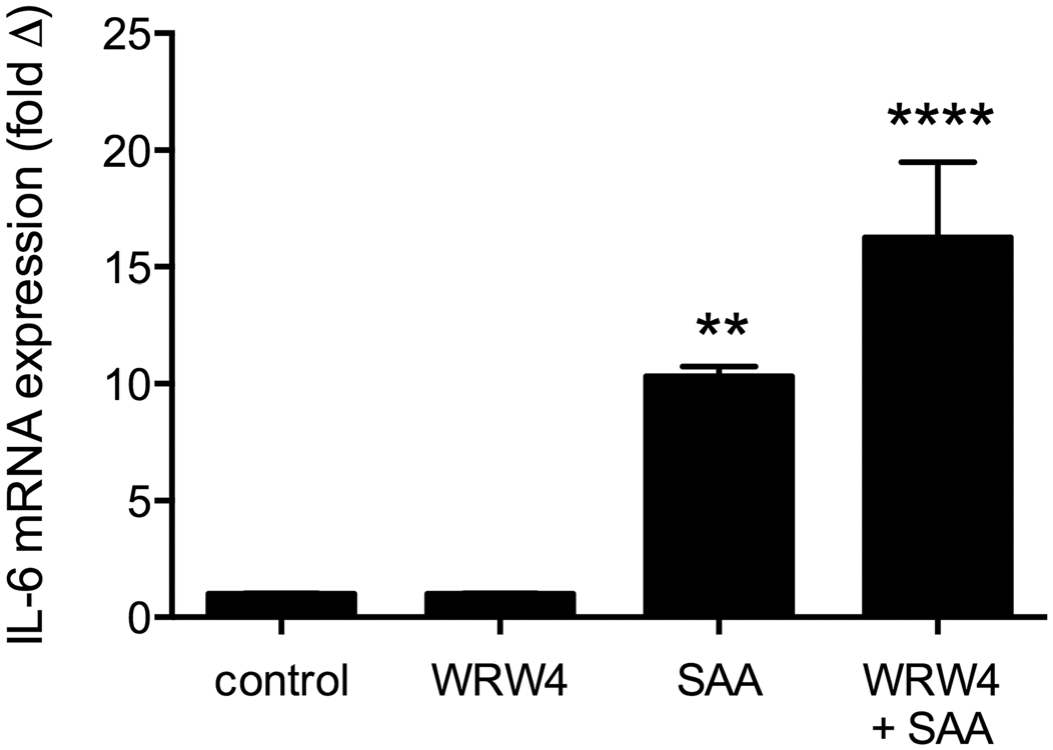
Treatment with WRW4 did not reduce IL-6 expression in myotubes stimulated with SAA. Myotubes were pre-treated for 20 minutes with WRW4 (5.5μg/ml) prior to stimulation with SAA (1 μg/ml) for 3h. IL-6 mRNA levels were normalised to 18s rRNA and expressed relative to control untreated sample. Data shown are mean ± SEM from n = 3 independent experiments, ** p<0.01, **** p<0.0001 vs. control.

Despite the low expression of fpr2 by qRT-PCR in the C2C12 myotubes others have observed responses to the fpr2 ligand Annexin A1 in these cells [35], prompting further investigation into whether SAA could be acting through fpr2 to induce pro-inflammatory cytokine expression. Myotubes were incubated with the fpr2 antagonist WRW4 for 20 minutes prior to treatment with a submaximal concentration of SAA (1μg/ml) for 3 hours. Gene expression analysis revealed significant increases in IL-6 transcription in cells treated with SAA alone and with WRW4 + SAA, however no significant difference in IL-6 expression was seen between cells treated with SAA alone and those treated with WRW4 and SAA (Fig 3). These data suggest that at the concentration of WRW4 used SAA is acting via one of the alternative receptors and not through fpr2 to induce pro-inflammatory cytokine expression in these cells.

SAA has been reported to act via TLR2 [30, 36] and TLR4 [32, 33] in various cell types. LPS is a known ligand for TLR4, and trace contamination of recombinant SAA peptide with LPS could be a possible source of the cytokine responses we observed. The SAA peptide is reported to contain less than 0.1ng/μg of LPS, however to further confirm that the effects we observed in C2C12 were not due to LPS contamination, myotubes were treated with SAA pre-incubated with Polymyxin B, which binds LPS and prevents it from signalling via TLR4. There was no significant difference between cells stimulated with SAA in the presence or absence of Polymyxin B (see S1 Fig), confirming that the responses observed were due to SAA itself and not due to LPS contamination.

**Fig 4:**
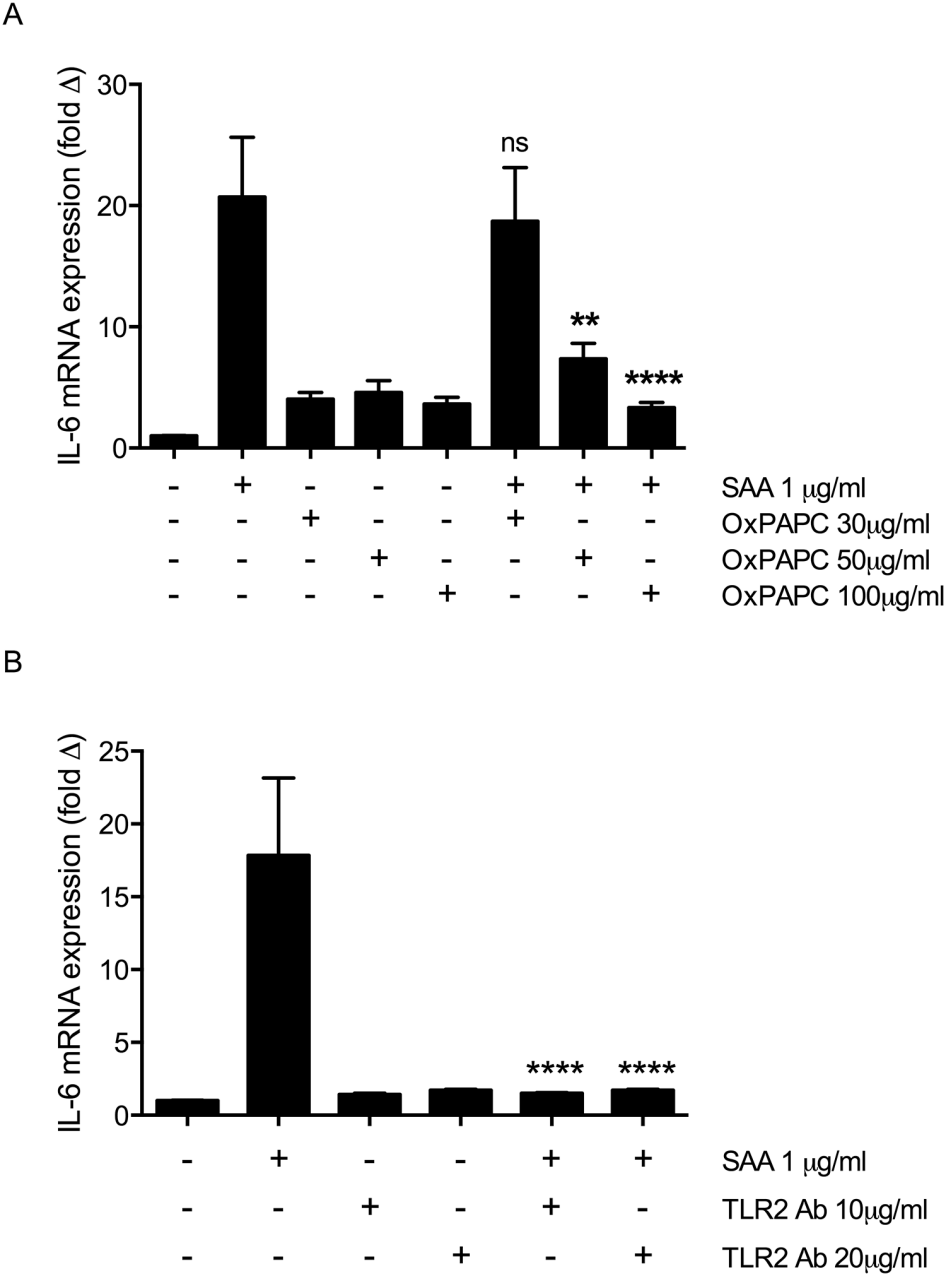
Blocking of TLR2 can prevent the SAA-induced increase in IL-6 transcription in C2C12 myotubes. (A) The effect of TLR2/4 inhibition with OxPAPC or (B) TLR2 neutralising antibody on IL-6 mRNA levels in SAA-treated myotubes. mRNA levels normalised to 18s rRNA and expressed relative to control untreated samples. Data shown are mean ± SEM for n = 3 independent experiments, statistical significance expressed vs SAA, ns = not significant, ** p<0.01, **** p< 0.0001.

SAA signalling via TLR2 and TLR4 was further investigated by treating the cells with the TLR2/4 inhibitor OxPAPC [37] followed by SAA stimulation. A significant reduction in IL-6 mRNA expression was observed in response to SAA stimulation in cells treated with OxPAPC at 50 and 100 μg/ml, compared to SAA alone (p<0.01, Fig 4A). No significant difference was observed in cells treated with OxPAPC at 30 μg/ml. These results indicate that SAA is at least in part exerting its effects on pro-inflammatory cytokine expression via TLR2/4 signalling.

Treatment of myotubes with the neutralising TLR2 antibody at 10 or 20μg/ml for 1 hour prior to the addition of 1μg/ml SAA resulted in abolishment of the IL-6 transcriptional stimulation by SAA (p<0.0001, Fig 4B). This confirms that in C2C12 myotubes SAA is primarily acting via TLR2 to stimulate robust pro-inflammatory cytokine expression.

### SAA causes reduced myotube width in C2C12

**Fig 5:**
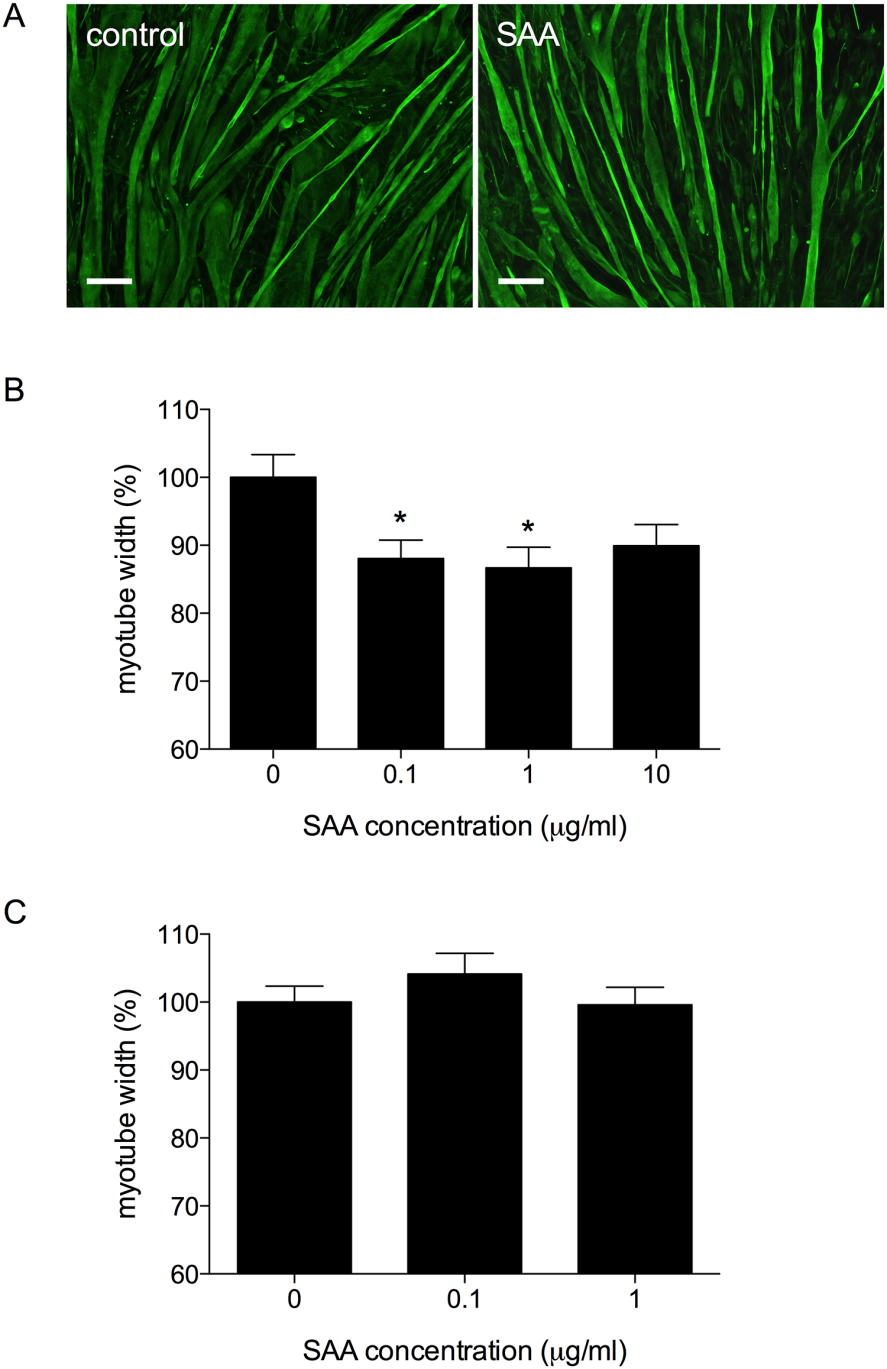
SAA causes reduction in myotube widths that can be reversed by blocking TLR2. (A) Control (left panel) and SAA 1μg/ml (right panel) C2C12 myotubes treated with SAA for 48h, immunolabelled with anti-desmin antibody (green). Scale bar = 100μm. (B) Quantification of myotube widths from immunofluorescence images. (C) Myotube widths in C2C12 myotubes treated with TLR2 neutralising antibody prior to SAA stimulation for 48h. Data shown are mean ± SEM for n = 3 independent experiments, statistical significance shown relative to control myotubes (0μg/ml SAA), * p<0.05.

We next investigated the effect of SAA treatment on myotube atrophy. Differentiated C2C12 myotubes were treated with SAA at 0.1, 1 and 10μg/ml for 48h, fixed and immunolabelled for the myogenic marker desmin (Fig 5A). SAA caused a small but significant reduction in myotube width at both 0.1μg/ml and 1μg/ml (Fig 5B p<0.05) compared to control untreated myotubes, equating to an approximately 10–13% reduction in myotube width in SAA treated cells.

To determine if SAA-induced atrophy could be mediated via TLR2, myotubes were incubated with the TLR2 neutralising antibody (10μg/ml) for 1 hour prior to stimulation with SAA at 0.1 and 1μg/ml for 48h. Myotube width was quantified as previously described. In contrast to myotubes treated with SAA alone, myotubes treated with SAA in the presence of the TLR2 antibody did not show significant levels of atrophy (Fig 5C). Blocking of TLR2 prevented the SAA-induced atrophy in C2C12 myotubes, indicating that SAA is likely acting via TLR2 to induce an atrophy response in these cells.

### Upregulation of the atrophy gene Atrogin-1 in C2C12 myotubes in response to SAA treatment

**Fig 6:**
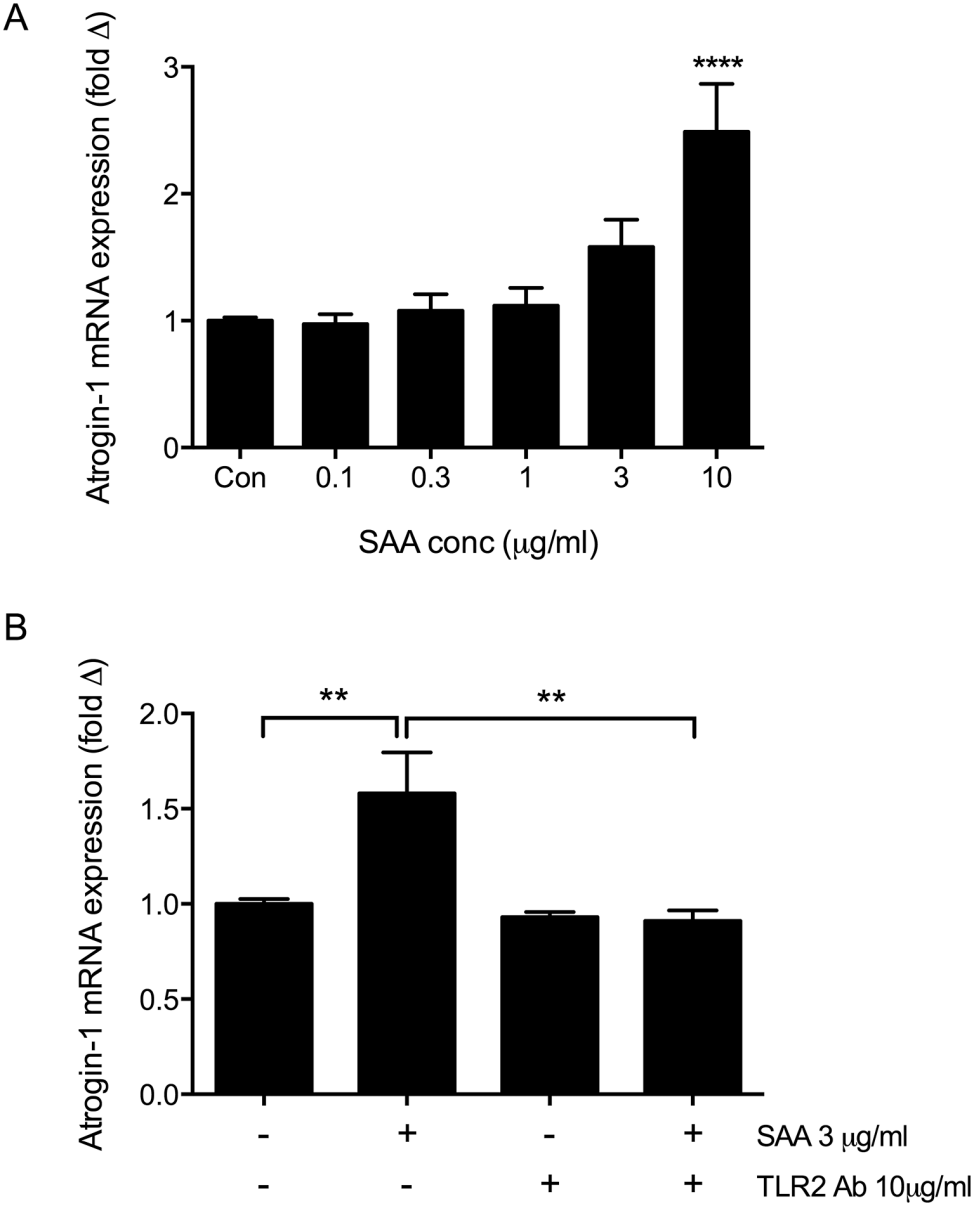
SAA induces Atrogin-1 mRNA expression in C2C12 myotubes that can be inhibited by blocking TLR2. (A) Atrogin-1 expression at 2h in the presence of varying concentrations of SAA. (B) Myotubes were pre-treated with TLR2 antibody (10μg/ml) for 1 hour prior to stimulation with SAA at 3μg/ml for 2h. Atrogin-1 mRNA was normalised to 18s rRNA and expressed relative to control. All data shown are mean ± SEM from n = 3 independent experiments, statistical significance shown relative to control unless otherwise indicated, **** p<0.0001, ** p<0.01.

Quantitative RT-PCR analysis of myotubes following SAA treatment revealed a significant increase in the expression of the muscle-specific E3 ligase Atrogin-1 after 2h of SAA stimulation at 10 μg/ml (Fig 6A, p<0.0001). Pre-treatment of myotubes with 10 μg/ml of TLR2 antibody prior to stimulation with a submaximal concentration of SAA (3μg/ml) effectively inhibited SAA-induced Atrogin-1 expression at 2h (Fig 6B), indicating that Atrogin-1 expression in response to SAA is mediated via TLR2.

## Discussion

In this study we sought to determine the effects of SAA on skeletal muscle myotubes using the C2C12 muscle cell line that is a widely used as an *in vitro* model of skeletal muscle [28, 38, 39].

Expression of genes for pro-inflammatory cytokines IL-6 and TNFα revealed a marked and robust dose-dependent increase in C2C12 myotubes after only 3h of SAA stimulation. Initial reports focused on the effects of SAA in immune cells such as neutrophils and macrophages [32, 40]. However recent studies have reported stimulation of IL-6 expression by SAA in other cell types including synoviocytes [41], dermal fibroblasts [31], and endothelial cells [42]. The level of secreted IL-6 protein from SAA-stimulated myotubes is comparable to that reported by others in response to LPS or Pam3CSK4, a TLR2 agonist [28]. These results indicate that C2C12 are capable of mounting a robust pro-inflammatory response following SAA treatment. This is a novel finding that has implications for many pathological conditions that exhibit high circulating SAA levels where skeletal muscle could potentially be a significant contributor towards the pro-inflammatory state.

SAA has been reported to bind a number of receptors including fpr2, TLR2, TLR4 and the scavenger receptor CD36 [29–34]. In control myotubes TLR2, TLR4 and CD36 were abundantly expressed, whilst fpr2 was detected at a much lower abundance. Expression of TLR2 mRNA was dramatically increased by approximately 30-fold following SAA stimulation. This is consistent with other studies showing that expression of TLR2 is upregulated in cells stimulated with the TLR4 ligand LPS or the TLR2 ligand Pam3CSK4 [43], a response proposed to be mediated by NFκB signalling.

Levels of fpr2 mRNA also increased by approximately 5-fold following SAA stimulation. Expression of human FPR2/ALX is known to be regulated by both LPS (via TLR4) and IFN-γ [44]. In addition, mouse fpr2 expression is upregulated in response to TLR2 signalling in microglia [45]; it is therefore likely that fpr2 expression is being regulated by SAA via TLR signalling through a similar mechanism in C2C12 myotubes. The upregulation of fpr2 and TLR2 following SAA stimulation could represent a priming mechanism within the muscle environment to allow for further stimulation by other inflammatory mediators, so potentiating the inflammatory response.

Treatment of cells with the fpr2 antagonist WRW4 [46] did not decrease the IL-6 transcriptional response of the myotubes to SAA stimulation, indicating that SAA is unlikely to be acting through fpr2 to stimulate IL-6 expression in these cells. However fpr2 is a complex receptor with numerous downstream signalling pathways [47], and it is possible that SAA is acting through fpr2 in C2C12 to stimulate other cellular responses not measured here.

The SAA induced IL-6 transcription response was reduced in a concentration dependent manner by the TLR2/4 inhibitor OxPAPC, and abolished by treatment with a neutralising TLR2 antibody. These findings indicate that in C2C12 myotubes SAA is primarily acting via TLR2 to initiate pro-inflammatory transcriptional responses. TLR2 has also been reported as a receptor for SAA in dermal fibroblasts [31], macrophages [30, 36], and coronary artery endothelial cells [42]. TLR2 is important in the recognition of pathogen or tissue-damage derived molecules as part of the innate immune response, and binds a variety of endogenous molecules called Damage Associated Molecular Patterns (DAMPs) released during infection, inflammation or as a result of tissue damage [48]. SAA is proposed to act as a DAMP and to signal through TLR2 to stimulate or promote the innate immune response. Our finding that SAA stimulates pro-inflammatory cytokine production in skeletal muscle myotubes via TLR2 further emphasises the role of SAA as a DAMP in the immune response, and highlights the often underestimated contribution of skeletal muscle to innate immunity and chronic inflammation.

Myotube width was significantly reduced in myotubes treated with SAA, with the most pronounced effect in myotubes treated with 1μg/ml SAA where a 13% decrease in myotube width was observed. This result contrasts with that reported by Zhang *et al*., who did not observe atrophy in L6 rat myotubes in response to SAA stimulation alone [20]. This is likely due to methodological differences including cell type and SAA treatment duration; we treated myotubes for 48h for microscopy analysis of myotube widths, compared to only 16h used by Zhang *et al*., which may not have been long enough for significant atrophy changes to occur. The SAA-induced atrophy response was inhibited by treatment with the TLR2 antibody, indicating that the myotube atrophy in response to SAA was mediated through TLR2.

One pathway that regulates muscle atrophy is the Ubiquitin proteasome pathway, and a number of muscle-specific E3-ubiquitin ligases mediate protein degradation in skeletal muscle through this pathway [49]. The E3 ligase Atrogin-1 was significantly increased in myotubes after SAA stimulation and this was inhibited by treatment with the TLR2 antibody, confirming that SAA regulates the expression of Atrogin-1 via TLR2. However, although we saw reductions in myotube widths in cells treated with 0.1 and 1μg/ml, Atrogin-1 expression was not significantly increased at these concentrations, suggesting the involvement of other mechanisms in the atrophy response observed in the myotubes. Others [50] have shown that insulin-like Growth Factor 1 (IGF-1) can inhibit Atrogin-1 expression in response to TNFα in C2C12 myotubes without reducing myotube atrophy, indicating that atrophy can occur through alternative pathways even if increases in Atrogin-1 are prevented.

Maintenance of muscle mass is achieved through the coordinated regulation of protein synthesis and protein breakdown through various interlinked molecular signalling pathways, many of which can be activated by multiple upstream receptors. Both TLR2 and fpr2 can signal via a number of downstream pathways to influence cellular processes and gene expression, including c-Jun N-terminal Kinase (JNK), p38 Mitogen Activated Kinase (p38 MAPK) and NFκB [47, 51]. Whilst inhibition of TLR2 is sufficient to reduce SAA-stimulated Atrogin-1 expression to baseline levels, SAA could also be acting in parallel through fpr2 to lead to atrophy through alternative signalling pathways.

Pro-inflammatory cytokines such as IL-6 can induce muscle atrophy *in vivo* [20, 52], although the effects of IL-6 are somewhat controversial and appear to be concentration and context dependent [53]. Although peak expression levels of both TNFα and IL-6 were stimulated by 3μg/ml SAA, expression was elevated above baseline with lower concentrations of SAA and this may have contributed to the myotube atrophy observed at these concentrations. Whilst the data presented here show a TLR2-dependent upregulation of one aspect of the ubiquitin-proteasome pathway in response to SAA, it is likely that multiple signalling pathways and mechanisms are responsible for the atrophy observed and further investigation is warranted to fully determine the relative contribution of these mechanisms to the overall atrophy response.

In this study we have shown for the first time that skeletal muscle myotubes mount a robust inflammatory response to SAA stimulation and that SAA stimulates an atrophy response in C2C12 myotubes. Skeletal muscle makes up approximately 40% of body mass, therefore SAA-induced muscle cytokine production could contribute significantly to the elevated pro-inflammatory cytokine levels seen in AECOPD patients. The myotube atrophy in response to SAA treatment is important not only in AECOPD patients, but also in a range of other diseases where circulating SAA levels are elevated and muscle wasting is seen including cancer cachexia, rheumatoid arthritis and critical illness myopathy. Both cytokine transcriptional responses and atrophy responses were mediated through SAA interacting with TLR2, as blocking TLR2 inhibited these effects. Therapeutic intervention targeted at the TLR2 pathways could be beneficial for the prevention of SAA-induced muscle atrophy and inflammatory responses in a range of inflammatory conditions.

## Supporting Information

S1 Data Excel spreadsheet providing the raw data for each figure. (XLSX)

S1 Fig Polymyxin B did not prevent the SAA-induced increase in IL-6 expression in C2C12 myotubes. IL-6 mRNA normalised to 18s rRNA and expressed relative to control, data shown are mean ± SEM for n = 4 independent experiments. ns = not significant.
(TIFF)

## References

[1] Maltais F, Decramer M, Casaburi R, Barreiro E, Burelle Y, Debigare R, et al An official american thoracic society/european respiratory society statement: update on limb muscle dysfunction in chronic obstructive pulmonary disease. Am J Respir Crit Care Med. 2014;189(9):e15–62. 10.1164/rccm.201402-0373ST . 24787074 PMC4098112

[2] Decramer M, Gosselink R, Troosters T, Verschueren M, Evers G. Muscle weakness is related to utilization of health care resources in COPD patients. The European respiratory journal. 1997;10(2):417–23. . 9042643 10.1183/09031936.97.10020417

[3] Marquis K, Debigare R, Lacasse Y, LeBlanc P, Jobin J, Carrier G, et al Midthigh muscle cross-sectional area is a better predictor of mortality than body mass index in patients with chronic obstructive pulmonary disease. Am J Respir Crit Care Med. 2002;166(6):809–13. 10.1164/rccm.2107031 . 12231489

[4] Schols AM, Broekhuizen R, Weling-Scheepers CA, Wouters EF. Body composition and mortality in chronic obstructive pulmonary disease. The American journal of clinical nutrition. 2005;82(1):53–9. . 16002800 10.1093/ajcn.82.1.53

[5] Swallow EB, Reyes D, Hopkinson NS, Man WD, Porcher R, Cetti EJ, et al Quadriceps strength predicts mortality in patients with moderate to severe chronic obstructive pulmonary disease. Thorax. 2007;62(2):115–20. Epub 2006/11/09. thx.2006.062026 [pii] 10.1136/thx.2006.062026 17090575 PMC2111256

[6] Spruit MA, Gosselink R, Troosters T, Kasran A, Gayan-Ramirez G, Bogaerts P, et al Muscle force during an acute exacerbation in hospitalised patients with COPD and its relationship with CXCL8 and IGF-I. Thorax. 2003;58(9):752–6. Epub 2003/08/30. 12947130 10.1136/thorax.58.9.752 PMC1746817

[7] Ansari K, Keaney N, Taylor I, Burns G, Farrow M. Muscle weakness, health status and frequency of exacerbations in chronic obstructive pulmonary disease. Postgraduate medical journal. 2012;88(1041):372–6. 10.1136/postgradmedj-2011-130293 . 22388793

[8] Vilaro J, Ramirez-Sarmiento A, Martinez-Llorens JM, Mendoza T, Alvarez M, Sanchez-Cayado N, et al Global muscle dysfunction as a risk factor of readmission to hospital due to COPD exacerbations. Respiratory medicine. 2010;104(12):1896–902. 10.1016/j.rmed.2010.05.001 . 20541383

[9] Bozinovski S, Hutchinson A, Thompson M, Macgregor L, Black J, Giannakis E, et al Serum amyloid a is a biomarker of acute exacerbations of chronic obstructive pulmonary disease. Am J Respir Crit Care Med. 2008;177(3):269–78. 10.1164/rccm.200705-678OC . 18006888

[10] Langhans C, Weber-Carstens S, Schmidt F, Hamati J, Kny M, Zhu X, et al Inflammation-induced acute phase response in skeletal muscle and critical illness myopathy. PloS one. 2014;9(3):e92048 Epub 2014/03/22. 10.1371/journal.pone.0092048 PONE-D-13-46130 [pii]. 24651840 PMC3961297

[11] Kumon Y, Suehiro T, Hashimoto K, Nakatani K, Sipe JD. Local expression of acute phase serum amyloid A mRNA in rheumatoid arthritis synovial tissue and cells. The Journal of rheumatology. 1999;26(4):785–90. . 10229397

[12] Cho WC, Yip TT, Cheng WW, Au JS. Serum amyloid A is elevated in the serum of lung cancer patients with poor prognosis. Br J Cancer. 2010;102(12):1731–5. Epub 2010/05/27. 6605700 [pii] 10.1038/sj.bjc.6605700 20502455 PMC2883701

[13] Bozinovski S, Uddin M, Vlahos R, Thompson M, McQualter JL, Merritt AS, et al Serum amyloid A opposes lipoxin A(4) to mediate glucocorticoid refractory lung inflammation in chronic obstructive pulmonary disease. Proceedings of the National Academy of Sciences of the United States of America. 2012;109(3):935–40. 10.1073/pnas.1109382109 22215599 PMC3271884

[14] Bonetto A, Aydogdu T, Kunzevitzky N, Guttridge DC, Khuri S, Koniaris LG, et al STAT3 activation in skeletal muscle links muscle wasting and the acute phase response in cancer cachexia. PloS one. 2011;6(7):e22538 10.1371/journal.pone.0022538 21799891 PMC3140523

[15] Goodman MN. Tumor necrosis factor induces skeletal muscle protein breakdown in rats. Am J Physiol. 1991;260(5 Pt 1):E727–30. Epub 1991/05/01. . 2035628 10.1152/ajpendo.1991.260.5.E727

[16] Haddad F, Zaldivar F, Cooper DM, Adams GR. IL-6-induced skeletal muscle atrophy. J Appl Physiol (1985). 2005;98(3):911–7. Epub 2004/11/16. 01026.2004 [pii] 10.1152/japplphysiol.01026.2004 . 15542570

[17] Pinto-Plata VM, Mullerova H, Toso JF, Feudjo-Tepie M, Soriano JB, Vessey RS, et al C-reactive protein in patients with COPD, control smokers and non-smokers. Thorax. 2006;61(1):23–8. Epub 2005/09/07. thx.2005.042200 [pii] 10.1136/thx.2005.042200 16143583 PMC2080714

[18] Broekhuizen R, Wouters EF, Creutzberg EC, Schols AM. Raised CRP levels mark metabolic and functional impairment in advanced COPD. Thorax. 2006;61(1):17–22. 10.1136/thx.2005.041996 16055618 PMC2080712

[19] Yende S, Waterer GW, Tolley EA, Newman AB, Bauer DC, Taaffe DR, et al Inflammatory markers are associated with ventilatory limitation and muscle dysfunction in obstructive lung disease in well functioning elderly subjects. Thorax. 2006;61(1):10–6. Epub 2005/11/15. thx.2004.034181 [pii] 10.1136/thx.2004.034181 16284220 PMC2080698

[20] Zhang L, Du J, Hu Z, Han G, Delafontaine P, Garcia G, et al IL-6 and serum amyloid A synergy mediates angiotensin II-induced muscle wasting. Journal of the American Society of Nephrology: JASN. 2009;20(3):604–12. 10.1681/ASN.2008060628 19158350 PMC2653674

[21] Kisilevsky R, Manley PN. Acute-phase serum amyloid A: perspectives on its physiological and pathological roles. Amyloid: the international journal of experimental and clinical investigation: the official journal of the International Society of Amyloidosis. 2012;19(1):5–14. 10.3109/13506129.2011.654294 . 22320226

[22] Stevenson EJ, Koncarevic A, Giresi PG, Jackman RW, Kandarian SC. Transcriptional profile of a myotube starvation model of atrophy. J Appl Physiol (1985). 2005;98(4):1396–406. 10.1152/japplphysiol.01055.2004 . 15608089

[23] Trendelenburg AU, Meyer A, Rohner D, Boyle J, Hatakeyama S, Glass DJ. Myostatin reduces Akt/TORC1/p70S6K signaling, inhibiting myoblast differentiation and myotube size. American journal of physiology Cell physiology. 2009;296(6):C1258–70. 10.1152/ajpcell.00105.2009 . 19357233

[24] Hughes DC, Stewart CE, Sculthorpe N, Dugdale HF, Yousefian F, Lewis MP, et al Testosterone enables growth and hypertrophy in fusion impaired myoblasts that display myotube atrophy: deciphering the role of androgen and IGF-I receptors. Biogerontology. 2015 10.1007/s10522-015-9621-9 . 26538344 PMC4889645

[25] Livak KJ, Schmittgen TD. Analysis of relative gene expression data using real-time quantitative PCR and the 2(-Delta Delta C(T)) Method. Methods. 2001;25(4):402–8. . 11846609 10.1006/meth.2001.1262

[26] Blau HM, Pavlath GK, Hardeman EC, Chiu CP, Silberstein L, Webster SG, et al Plasticity of the differentiated state. Science. 1985;230(4727):758–66. Epub 1985/11/15. . 2414846 10.1126/science.2414846

[27] Sharples AP, Al-Shanti N, Stewart CE. C2 and C2C12 murine skeletal myoblast models of atrophic and hypertrophic potential: relevance to disease and ageing? Journal of cellular physiology. 2010;225(1):240–50. 10.1002/jcp.22252 . 20506232

[28] Frost RA, Nystrom GJ, Lang CH. Multiple Toll-like receptor ligands induce an IL-6 transcriptional response in skeletal myocytes. American journal of physiology Regulatory, integrative and comparative physiology. 2006;290(3):R773–84. 10.1152/ajpregu.00490.2005 . 16254126

[29] Su SB, Gong W, Gao JL, Shen W, Murphy PM, Oppenheim JJ, et al A seven-transmembrane, G protein-coupled receptor, FPRL1, mediates the chemotactic activity of serum amyloid A for human phagocytic cells. The Journal of experimental medicine. 1999;189(2):395–402. 9892621 10.1084/jem.189.2.395 PMC2192984

[30] Cheng N, He R, Tian J, Ye PP, Ye RD. Cutting edge: TLR2 is a functional receptor for acute-phase serum amyloid A. Journal of immunology. 2008;181(1):22–6. 18566366 10.4049/jimmunol.181.1.22 PMC2464454

[31] O'Reilly S, Cant R, Ciechomska M, Finnegan J, Oakley F, Hambleton S, et al Serum Amyloid A (SAA) induces IL-6 in dermal fibroblasts via TLR2, IRAK4 and NF-kappaB. Immunology. 2014 10.1111/imm.12260 . 24476318 PMC4212947

[32] Sandri S, Rodriguez D, Gomes E, Monteiro HP, Russo M, Campa A. Is serum amyloid A an endogenous TLR4 agonist? Journal of leukocyte biology. 2008;83(5):1174–80. 10.1189/jlb.0407203 . 18252871

[33] Tamamoto T, Ohno K, Goto-Koshino Y, Tsujimoto H. Feline serum amyloid A protein as an endogenous Toll-like receptor 4 agonist. Veterinary immunology and immunopathology. 2013;155(3):190–6. 10.1016/j.vetimm.2013.06.010 . 23942262

[34] Baranova IN, Bocharov AV, Vishnyakova TG, Kurlander R, Chen Z, Fu D, et al CD36 is a novel serum amyloid A (SAA) receptor mediating SAA binding and SAA-induced signaling in human and rodent cells. The Journal of biological chemistry. 2010;285(11):8492–506. 10.1074/jbc.M109.007526 20075072 PMC2832998

[35] Bizzarro V, Belvedere R, Dal Piaz F, Parente L, Petrella A. Annexin A1 induces skeletal muscle cell migration acting through formyl peptide receptors. PloS one. 2012;7(10):e48246 10.1371/journal.pone.0048246 23144744 PMC3483218

[36] He RL, Zhou J, Hanson CZ, Chen J, Cheng N, Ye RD. Serum amyloid A induces G-CSF expression and neutrophilia via Toll-like receptor 2. Blood. 2009;113(2):429–37. 10.1182/blood-2008-03-139923 18952897 PMC2615655

[37] Erridge C, Kennedy S, Spickett CM, Webb DJ. Oxidized phospholipid inhibition of toll-like receptor (TLR) signaling is restricted to TLR2 and TLR4: roles for CD14, LPS-binding protein, and MD2 as targets for specificity of inhibition. The Journal of biological chemistry. 2008;283(36):24748–59. 10.1074/jbc.M800352200 18559343 PMC3259833

[38] Player DJ, Martin NR, Passey SL, Sharples AP, Mudera V, Lewis MP. Acute mechanical overload increases IGF-I and MMP-9 mRNA in 3D tissue-engineered skeletal muscle. Biotechnology letters. 2014;36(5):1113–24. 10.1007/s10529-014-1464-y . 24563297

[39] Sharples AP, Stewart CE. Myoblast models of skeletal muscle hypertrophy and atrophy. Current opinion in clinical nutrition and metabolic care. 2011;14(3):230–6. 10.1097/MCO.0b013e3283457ade . 21460719

[40] Furlaneto CJ, Campa A. A novel function of serum amyloid A: a potent stimulus for the release of tumor necrosis factor-alpha, interleukin-1beta, and interleukin-8 by human blood neutrophil. Biochem Biophys Res Commun. 2000;268(2):405–8. . 10679217 10.1006/bbrc.2000.2143

[41] Koga T, Torigoshi T, Motokawa S, Miyashita T, Maeda Y, Nakamura M, et al Serum amyloid A-induced IL-6 production by rheumatoid synoviocytes. FEBS letters. 2008;582(5):579–85. 10.1016/j.febslet.2008.01.022 . 18243142

[42] Lakota K, Mrak-Poljsak K, Bozic B, Tomsic M, Sodin-Semrl S. Serum amyloid A activation of human coronary artery endothelial cells exhibits a neutrophil promoting molecular profile. Microvascular research. 2013;90:55–63. 10.1016/j.mvr.2013.07.011 . 23938271

[43] van Aubel RA, Keestra AM, Krooshoop DJ, van Eden W, van Putten JP. Ligand-induced differential cross-regulation of Toll-like receptors 2, 4 and 5 in intestinal epithelial cells. Molecular immunology. 2007;44(15):3702–14. 10.1016/j.molimm.2007.04.001 . 17493681

[44] Waechter V, Schmid M, Herova M, Weber A, Gunther V, Marti-Jaun J, et al Characterization of the promoter and the transcriptional regulation of the lipoxin A4 receptor (FPR2/ALX) gene in human monocytes and macrophages. Journal of immunology. 2012;188(4):1856–67. Epub 2012/01/17. jimmunol.1101788 [pii] 10.4049/jimmunol.1101788 . 22246625

[45] Chen K, Zhang L, Huang J, Gong W, Dunlop NM, Wang JM. Cooperation between NOD2 and Toll-like receptor 2 ligands in the up-regulation of mouse mFPR2, a G-protein-coupled Abeta42 peptide receptor, in microglial cells. Journal of leukocyte biology. 2008;83(6):1467–75. Epub 2008/02/27. jlb.0907607 [pii] 10.1189/jlb.0907607 . 18299458

[46] Shin EH, Lee HY, Kim SD, Jo SH, Kim MK, Park KS, et al Trp-Arg-Trp-Trp-Trp-Trp antagonizes formyl peptide receptor like 2-mediated signaling. Biochem Biophys Res Commun. 2006;341(4):1317–22. 10.1016/j.bbrc.2006.01.098 . 16476585

[47] Cattaneo F, Parisi M, Ammendola R. Distinct signaling cascades elicited by different formyl peptide receptor 2 (FPR2) agonists. International journal of molecular sciences. 2013;14(4):7193–230. 10.3390/ijms14047193 23549262 PMC3645683

[48] Kawai T, Akira S. The role of pattern-recognition receptors in innate immunity: update on Toll-like receptors. Nature immunology. 2010;11(5):373–84. 10.1038/ni.1863 . 20404851

[49] Bodine SC, Latres E, Baumhueter S, Lai VK, Nunez L, Clarke BA, et al Identification of ubiquitin ligases required for skeletal muscle atrophy. Science. 2001;294(5547):1704–8. 10.1126/science.1065874 . 11679633

[50] Dehoux M, Gobier C, Lause P, Bertrand L, Ketelslegers JM, Thissen JP. IGF-I does not prevent myotube atrophy caused by proinflammatory cytokines despite activation of Akt/Foxo and GSK-3beta pathways and inhibition of atrogin-1 mRNA. Am J Physiol Endocrinol Metab. 2007;292(1):E145–50. 10.1152/ajpendo.00085.2006 . 16926385

[51] O'Neill LA, Golenbock D, Bowie AG. The history of Toll-like receptors—redefining innate immunity. Nat Rev Immunol. 2013;13(6):453–60. Epub 2013/05/18. nri3446 [pii] 10.1038/nri3446 . 23681101

[52] Goodman MN. Interleukin-6 induces skeletal muscle protein breakdown in rats. Proc Soc Exp Biol Med. 1994;205(2):182–5. Epub 1994/02/01. . 8108469 10.3181/00379727-205-43695

[53] Munoz-Canoves P, Scheele C, Pedersen BK, Serrano AL. Interleukin-6 myokine signaling in skeletal muscle: a double-edged sword? FEBS J. 2013;280(17):4131–48. Epub 2013/05/15. 10.1111/febs.12338 23663276 PMC4163639

