# Supplementary figures and images for: Serum Amyloid A Induces Toll-Like Receptor 2-Dependent Inflammatory Cytokine Expression and Atrophy in C2C12 Skeletal Muscle Myotubes
Source: PLoS One. 2016 Jan 19;11(1):e0146882. doi: 10.1371/journal.pone.0146882 (PMC4718684; doi:10.1371/journal.pone.0146882)

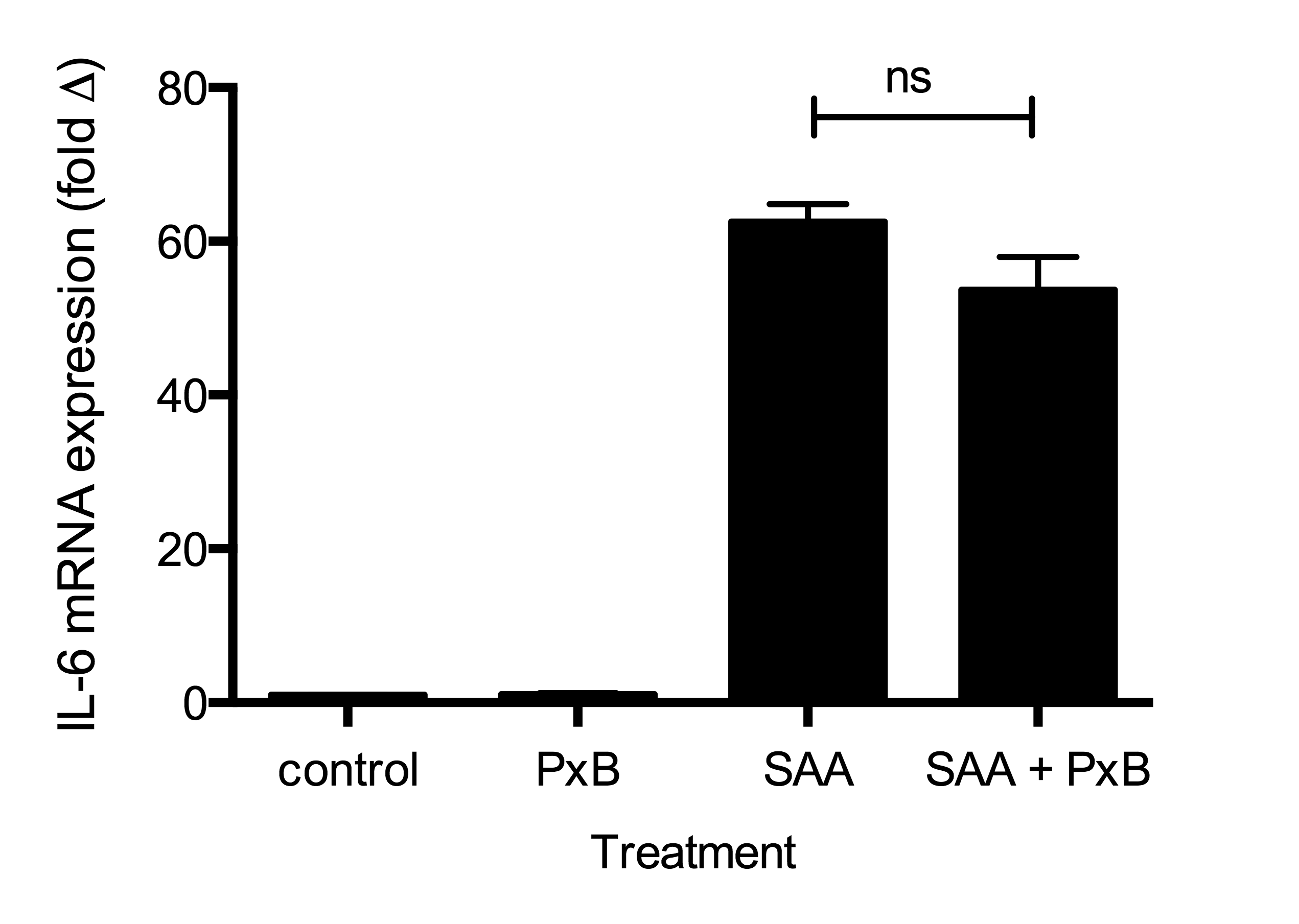

Supplement: S1 Fig — IL-6 mRNA normalised to 18s rRNA and expressed relative to control, data shown are mean ± SEM for n = 4 independent experiments. ns = not significant. (TIFF) [file pone.0146882.s002.tiff]
